# Simplifying healthful choices: a qualitative study of a physical activity based nutrition label format

**DOI:** 10.1186/1475-2891-12-72

**Published:** 2013-06-06

**Authors:** Jonas J Swartz, Sunaina Dowray, Danielle Braxton, Paul Mihas, Anthony J Viera

**Affiliations:** 1 Department of Obstetrics and Gynecology, Oregon Health and Science University, 3181 SW Sam Jackson Park Rd., L 466, Portland, OR 97239, USA; 2 University of North Carolina School of Medicine, Chapel Hill, NC, USA; 3 University of North Carolina Center for Health Promotion and Disease Prevention, Chapel Hill, NC, USA; 4 The Odum Institute, University of North Carolina at Chapel Hill, Chapel Hill, NC, USA; 5 Department of Family Medicine, University of North Carolina School of Medicine, and Public Health Leadership Program, University of North Carolina Gillings School of Global Public Health, Chapel Hill, NC, USA

**Keywords:** Calorie label, Menu label, Nutrition information, Restaurant label, Patient protection and affordable care act, Obesity, Food away from home, Fast food

## Abstract

**Background:**

This study used focus groups to pilot and evaluate a new nutrition label format and refine the label design. Physical activity equivalent labels present calorie information in terms of the amount of physical activity that would be required to expend the calories in a specified food item.

**Methods:**

Three focus groups with a total of twenty participants discussed food choices and nutrition labeling. They provided information on comprehension, usability and acceptability of the label. A systematic coding process was used to apply descriptive codes to the data and to identify emerging themes and attitudes.

**Results:**

Participants in all three groups were able to comprehend the label format. Discussion about label format focused on issues including gender of the depicted figure, physical fitness of the figure, preference for walking or running labels, and preference for information in miles or minutes. Feedback from earlier focus groups was used to refine the labels in an iterative process.

**Conclusions:**

In contrast to calorie labels, participants shown physical activity labels asked and answered, “How does this label apply to me?” This shift toward personalized understanding may indicate that physical activity labels offer an advantage over currently available nutrition labels.

## Introduction

Calorie labeling is one of many policy approaches proposed to address the high prevalence of overweight and obesity in the United States [1-3]. Current strategies in calorie labeling include efforts mainly to increase the visibility of labels at the point of purchase. The Patient Protection and Affordable Care Act of 2010 requires that all chain restaurants with 20 or more locations begin to list the calorie information in the foods and beverages they serve. This new legislation builds upon efforts already underway in some states to give consumers more information about the foods they purchase away from home [4]. However, this strategy does not have strong empirical justification. Two systematic reviews conducted in 2008 [5] and 2011 [6] identified six [7-10] and seven [11-16] studies, respectively, that evaluated the effects of calorie labels on consumer choice. Both reviews found only weak, inconsistent evidence that calorie menu labels lead consumers to make lower-calorie food choices [5,6].

Since more visible calorie labels may not be sufficient to influence choice, another strategy may be improving the usability of calorie labels. Specifically, labels that frame nutritional information in more familiar and tangible ways may be easier to understand and have greater potential to influence choice than those that provide only caloric data [17]. We designed and tested understanding and acceptability of labels that quantified calorie information in terms of energy balance; the labels depicted how much physical activity would be required to expend the calories in the food. The labels employed an iconographic design rather than text or text with graphics used in other studies [18,19]. The purpose of this study was to assess consumer comprehension of an energy balance label and evaluate the potential for such a label to influence consumer choice.

## Background

Health advocates, government regulators and the food industry are working on multiple approaches to increase visibility and usability of nutrition labels. Several strategies being put into practice include calorie menu labels, front-of-the-pack nutrition labels on prepackaged foods in grocery stores, and universal symbols designating healthy foods. Research on nutrition label use and understanding indicates that both vary considerably among different demographics [20]. In particular, consumers have difficulty contextualizing individual food choices within a total diet [21-24]. Even when nutrition information is available, consumers may value variables like taste and cravings more highly, and probably do not use nutritional information explicitly in most food choices [23]. Furthermore, even consumers concerned with nutrition may lack the self-control required to make the individual healthy choices necessary to achieve lifestyle change [21].

A few publications have suggested calorie labels with a physical activity equivalent might help consumers make lower calorie choices [17,21], and two studies have quantitatively assessed acceptability of a physical activity label format [19,25]. Bleich and Pollack found respondents to be equally divided in their preference for standard calorie labels, physical activity equivalent labels and percentage of total daily calories [25]. Fitch et al. piloted a physical activity equivalent label beside calorie information and found that a majority of participants preferred calorie information [19]. One study showed low-income adolescents purchased fewer sugar-sweetened beverages when exposed to physical activity equivalent labels [18].

Introducing an iconographic label requires special attention to comprehension and interpretation. Qualitative studies of pharmaceutical warning labels, for example, have shown that common interpretation of labels differs from what the label designers intend to convey [26,27]. Qualitative study of physical activity nutrition labels is limited. Van Kleef et al. conducted a relatively large, multi-country focus group study assessing consumer understanding of different formats and testing comprehension and usability of different designs [28]. They found that European consumers preferred other types of labels to exercise labels as they found the labels “demotivating and patronising [28].” Though consumers understood multiple label formats, van Kleef, et al. noted participants had difficulty using calorie information to contextualize individual food choices in overall daily diet [28]. Specifically, users were not sure of their personal daily caloric needs even when prompted with general information about daily caloric needs [28].

## Methods

### Study design

We used focus groups to explore preference regarding various label designs, gauge understanding of their content, and assess their potential for influencing food decisions. We chose to field three focus groups as three sessions allowed us to modify the questions and further develop the labels. Study procedures and the focus group guide received approval from the Institutional Review Board (IRB) at the University of North Carolina at Chapel Hill (UNC). We received funding through the University Research Council at UNC.

### Study location and participants

We conducted three focus groups in Chapel Hill, North Carolina. The site provided good access to a socially, ethnically and demographically diverse population.

Participants were recruited through convenience sampling. We contacted healthy volunteers from an IRB-approved list of previous research participants maintained by one investigator. We also sent campus-wide invitational emails to UNC students, faculty and staff, and posted flyers at the UNC Family Medicine Center, the UNC Clinical and Translational Research Center and community locations including grocery stores, gyms and churches.

To qualify, participants had to be 25 years or older in order to get feedback from participants who had more substantial experience shopping in grocery stores and eating at quick-service restaurants. Participants had to have shopped in a grocery store in the past month and purchased food from a restaurant in the past two weeks. Exclusion criteria included living in a dormitory, non-English speaking and prior special training in nutrition. A questionnaire administered by phone was used to establish eligibility.

We had eight participants in the first focus group session and six participants in the second and third sessions. All participants who arrived for the focus group received a $20 gift card, and we served lunch to participants.

### Focus group discussions

The focus groups were facilitated by two investigators with one investigator acting as moderator and one other observing and taking detailed notes. The moderator’s guide was developed through collaboration of the study investigators, who sought additional feedback from colleagues with expertise in qualitative research methods (Appendix 1). On arrival, participants completed a questionnaire on demographic information and the Newest Vital Sign, a rapid health literacy assessment [29].

Focus group discussions lasted a total of 90 minutes and were held at midday on a weekend. After an icebreaker, participants were asked to describe how they make decisions about which foods to choose, and whether they pay attention to nutrition or healthfulness of their diets. They then were asked specifically to explain their rationale for making a food choice in a quick-service restaurant.

**Figure 1:**
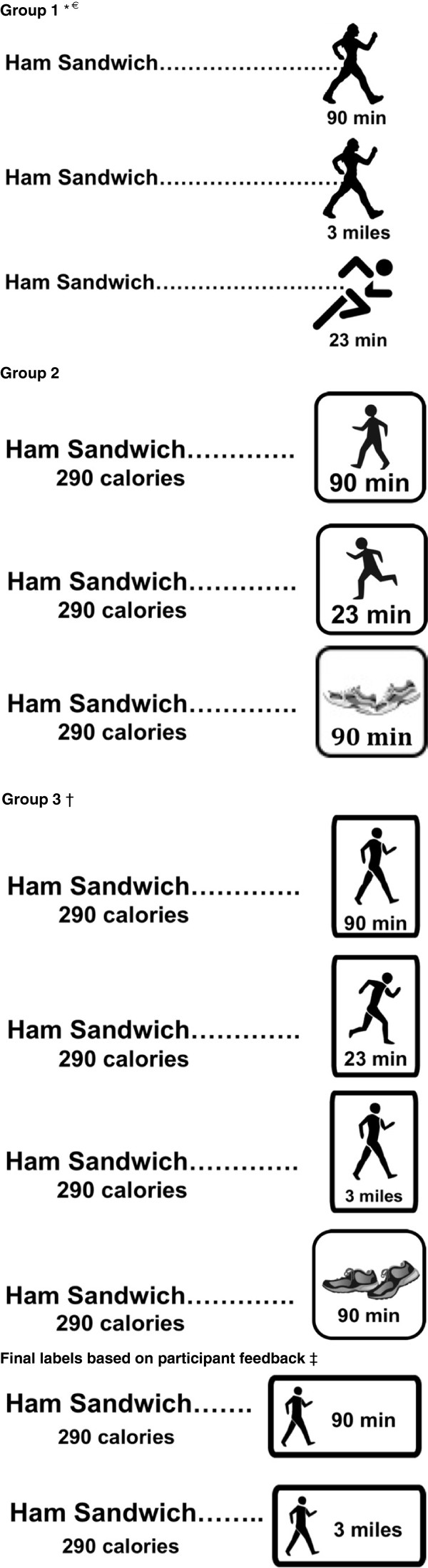
**Sample labels used for focus groups.** * Illustrations from http://www.silhouettesclipart.com/walking-silhouette-clip-art.html, ^€^ Illustration from http://student.ucr.edu/~schav014/running.jpg, ^†^ Illustrations by Alicia Orth, ^‡^ Illustrations and label design by Clay Braxton.

We then offered participants a choice of sandwiches and beverages for lunch. Calorie labels in several formats (Figure 1) were displayed via PowerPoint giving caloric information about the sandwiches and beverages being served for lunch. By providing labels for the food available at the focus group, we did not intend to influence participant choices or eating with the labels, but rather to make the labels easy to relate to and contextually appropriate. We asked participants to compare physical activity labels with a label stating the number of calories and their contribution to the recommended daily allowance. Participants were asked to describe what each of the label formats meant. They were also asked how they might change the label. In the context of the discussion on label refinements, participants were shown iterations of labels with slight variations, for example expressing the amount of physical activity in mileage rather than time, changing the picture on the label, changing the font, and altering the wording (Figure 1). Feedback from prior focus group discussions was used to adapt the label design for the second and third focus groups.

Next, participants were asked whether and how they thought the labels might influence their food choices when at a quick-service restaurant. They were then asked to talk about the potential role for these labels for prepackaged food products like cereal, and whether they would use them. Finally, participants had the opportunity to talk about any food labeling issues we had not covered in the focus groups.

### Label design

We designed a variety of menu labels in consultation with experts in nutrition and medical illustration. We refined the labels in an iterative process, using feedback from early focus groups to guide label development throughout the study. Many variables including body weight, gender, age and basal metabolic rate affect energy expenditure for individuals. Since we sought to produce a universally applicable label, we simplified by excluding some of these variables. To calculate average energy expenditure for labels depicting running or walking, we used an average body weight of 160 pounds. We used an energy expenditure chart that listed estimated calories burned by activity and body weight [30]. For labels depicting walking, we used the energy expenditure of a 160 pound adult walking at a rate of 30 minutes per mile (3.2 kcal/min). For running, we used the energy expenditure of a 160 pound adult running at a rate of 10 minutes per mile (12.8 kcal/min). To determine the number of minutes required to burn off calories in a food item, we divided the total calories in the item by the energy expenditure rate. To calculate the number of miles that would be required to expend the calories in a food item, we divided the total time required by the running or walking pace. Sample calculations are included (Appendix 2). We obtained information on the caloric content of food and beverage items from the company websites [31,32].

Using feedback from prior groups, we were able to adapt the labels for the second and third groups to meet participant preferences. Each set of labels is included (Figure 1).

### Analysis

Focus group conversations were recorded using a digital voice recorder and transcribed into a Microsoft Word file. Transcripts were compared with detailed notes taken by the assistant moderator during the sessions to verify their accuracy and clarify any instances where the transcriber was not able to identify the speaker. The moderator and assistant moderator reviewed the transcripts together.

Once the transcripts were finalized, we used ATLAS.ti Qualitative Data Analysis software in consultation with a qualitative data analysis expert who did not take part in the planning or focus groups. We systematically coded the data using descriptive codes and identified emerging themes and attitudes. We used representative verbatim statements in our analysis. Quantitative data including group demographic characteristics were analyzed in Microsoft Excel.

## Results

Thirty-eight potential participants were contacted for the study and 34 met inclusion and exclusion criteria. We fielded three focus groups with eight participants in the first group and six in each of the subsequent groups. Over half (65%) of participants were female and 45% were white (Table 1). More than half (65%) of participants lived with a significant other, less than half (35%) lived with children, and the majority of participants (90%) had adequate health literacy (Table 2).

**Table 1:** Demographic characteristics of focus group participants

	***%***	***N***
*Gender*		
Female	65	13
Male	35	7
Race*		
African-American	45	9
American Indian	5	1
Asian/Pacific Islander	10	2
Caucasian	45	9
Latino/Hispanic	5	1
*Age*		
25-40	35	7
41-60	45	9
>60	20	4
*Education*		
< High school	0	0
High school graduate	5	1
Some college	40	8
College graduate or higher	55	11
*Household income*		
<$20,000	5	1
$20,000-$49,999	45	9
$50,000-$74,999	10	2
$75,000-100,000	15	3
>$100,000	25	5

*Participants self-identified and could mark multiple categories.

**Table 2:** Lifestyle habits and health literacy of study participants

	**%**	**N**
*Live with significant other*		
Yes	65	13
No	35	7
*Live with children*		
Yes	35	7
No	65	13
*Weekly visits to grocery store*		
0-1	0	0
2-3	80	16
>3	20	4
*Weekly visits to fast food restaurant*		
0-1	50	10
2-3	50	10
Health literacy*		
>50% chance of marginal or limited literacy	0	0
Possibly limited literacy	10	2
Adequate literacy	90	18

*Health literacy measured using the Newest Vital Sign, a rapid health literacy assessment.

### Food choices and nutritional information

Participants described a number of factors influencing their food choices including cravings, past experience with particular foods, price and nutrition or health concerns. Relating to nutrition, participants categorized healthfulness of foods in multiple ways including how fat and calorie contents related to their dietary goals, whether foods were natural or organic, and how many ingredients they had. Some used nutrition labels to look for sugar or sodium content. For example, one participant commented, “I tend to look not only at the sodium content, because it’s so high, [but also] at the sugar content. That can be helpful when I have wanted to lose a little weight and I was stuck.” Participants also reported that current nutrition label formats are often confusing. Common concerns include inability to reconcile individual food items in a daily dietary plan, specific confusion about the 2000 calorie daily diet that appears on many nutrition labels, and how to use labels to identify healthy foods. In one session, participants said that they like summary information like a green check or healthy heart that appears on some labels, but participants in that group and in others said they do not always trust companies to be honest about what foods are healthy. Several participants said they had seen “healthy” symbols attached to items they did not consider healthy. One person said, “I don’t [like the heart healthy label]. It might be healthy, but there’s a bunch of sugar and there’s a bunch of sodium. I would rather just have [information on] calories.”

In addition to questions of trust, participants said emotions like guilt, reward, unhappiness, defeat, burnout and nostalgia influence their food choices. For some, visiting a fast food restaurant can be a reward and cause them to disregard concerns about healthfulness. One participant said, “People think, ‘I deserve it today. I worked harder, I did something or I had more stress.’ Food is often used as a reward. You may look at it like, it’s gonna cost me a few extra calories, it’s gonna to cost me 20 more minutes here, but I’m worth it today.” More frequently, participants voiced guilt, unhappiness, defeat and burnout led them to eat things they knew to be unhealthy. “I feel less guilty when I try harder [to eat healthy] . . . But if I’m putting my all into it—I’m not even really trying to be a health freak—I’ll be unhappy. You know, every little thing you put in your mouth, everything you drink. I mean, it’ll just be too much. You can’t spend your whole life worrying about every little detail. Most people don’t even have that much time.”

### Participant understanding of labels

In all three focus groups, participants were able to verbally interpret iconographic labels. One participant put it this way: “To go back to zero, as if you’ve never had anything, as if you’d never eaten you have to walk this amount of time to burn it off.” As another participant put it, “It means you are going to have to do that much exercise to burn the calories you just ate.” One participant had an alternative interpretation: “Another way of looking at it would be that the Big Mac, with 540 calories, would be equivalent of providing you enough energy to walk 167 minutes.”

Though they were comfortable with the basic meaning of the labels, participants had questions about the activity depicted, the gender of the figure, the pace of walking or running depicted, and the apparent physical fitness of the figure compared to their own. All three focus groups devoted significant time and analysis to the personal applicability of the labels. One group was concerned with whether the labels would apply to children or physically disabled individuals. In another group, several participants debated whether the walking pace depicted should be for the average American, or only those who exercise.

The research team used details of the discussion and feedback from each group to develop labels to be tested with subsequent groups. However, the shift in conversation from calories as an abstract concept to personalized fitness assessment in relation to a label was perhaps more important. When interpreting numeric calorie labels, participants understood they applied to a particular item and even ranked items based on the label as “bad, good, better” in order of descending calorie count. Physical activity labels helped participants apply caloric information to their lives and thus their food choices. “What’s interesting to me is there’s only 60 calories between the top and the bottom sandwich, [but] the difference in the amount of exercise is significant. I mean it’s almost 20 minutes. To me, that’s a big difference. So you can think I might say gosh for just 60 calories less I can do 20 minutes less of exercise. So it would help inform my choice, I think.”

More tellingly, when presented with physical activity labels, each of the groups began a discussion of whether the labels were personally applicable and how they might be modified to apply to the average American. For example, several groups thought labels designed with information for an average weight of 160 pounds could be misleading or unrealistic and would need to be clarified. “Let’s just say that you show someone this nutrition label, would it have that disclaimer at the bottom that this is based on a 160 pound adult? Because that might not be me. [It should be there.] That way, I can calculate maybe my own number based on my weight.” The change in focus itself indicates that participants regarded physical activity labels differently.

### Participant preferences for label types

In designing the labels, we expected that more participants would identify with icons that depicted walking than those that depicted running. Participants said that walking is an activity that more people can do, so people may find the information more applicable to their lives. One participant expressed, “I would prefer walking only because the average person could walk. . . But running, everybody’s not going to run. So they’re going to look at it and be like whatever. I’m gonna be like, I’m not running, but I’ll eat this Big Mac.” Though it was a minority opinion, two participants said that, because they found the idea of running more intimidating, a running label might be more influential. “If your attempt here is to sound a warning that if you eat a Big Mac, here’s how you’re going to have to pay for it, [the running label] is more intimidating. It might dissuade you from having a Big Mac and get you to have a turkey sandwich in place of it.”

While depictions of walking were preferred over running depictions, participants were split on their preferences for information on mileage versus information on time of exercise required. Some said that they thought mileage was discouraging: “I think when you look at miles, it just seems like so much.” Others said they thought miles were a less common frame of reference. For example, one person commented, “We all understand what a minute is, but we may not understand what a mile is.” However, several participants preferred miles because people could move at their preferred pace and choose to walk or run.

### Unintended effects of labels

Participants reported that they might find the labels discouraging because they would not be able to carry out a particular physical activity, because the depicted activity is more than they do habitually, or because the time to burn off an item is so great that it is not worth doing exercise at all. Two groups also discussed whether the labels implied that people need to exercise sufficiently to account for all of the calories they consume. Some group members thought they should exercise to burn the calories they consume while others were able to articulate a more accurate understanding of daily caloric needs and basal metabolic rate. In general, as in the following quote, people said that they found the topic somewhat confusing. “You’re eating calories every day whether or not you exercise or not, so how many do you eat a day, and how many do you really need to burn off? That’s something that I don’t understand.”

## Discussion

To our knowledge, this is the first qualitative study assessing in-depth consumer understanding of a label that presents calories in terms of a physical activity equivalent. Our study provides a foundation for further development and testing of such labels in future research.

Calorie labels and point-of-purchase menu labeling have the potential to influence consumer choice provided that consumers value the nutritional content of what they eat and have the literacy and numeracy skills to understand and utilize the information. Stated differently, nutrition label efficacy depends on relevance and comprehension. The Affordable Care Act mandated implementation of calorie menu labels as one strategy to help reduce the prevalence of overweight and obesity. Current evidence suggests calorie menu labels are insufficient to achieve this goal [5,6], which may be a problem of relevance, comprehension or both.

A functional label format must overcome some of the barriers that limit effectiveness of current labeling strategies. Issues include problems with consumer literacy and numeracy as well as label accuracy, applicability and accessibility. Our labels couple a picture of a physical activity with minutes, hours or miles. A user should “read” the label as, “You would need to walk for 90 minutes to burn off the calories in this food item.” In each of the focus groups, participants accurately verbalized understanding of the labels.

Moreover, in contrast to calorie labels, participants shown physical activity labels asked and answered, “How does this label apply to me?” The fact that this question became a focus of discussion in all three groups is perhaps the most robust evidence from this study. If physical activity labels more effectively prompt users to consider how dietary choices affect them than currently available calorie labels, they may have greater potential to influence choice.

Details of the physical activity label were significant to participants because they compared the walking pace and metabolic rate used to calculate the label to their own. While it is important that users find the labels personally applicable, accuracy of the physical activity equivalent for an individual is actually less critical. As long as the labels accurately portray the difference between caloric content of various food items, consumers can use that information to make a choice. Prompting participation in physical activity would be an unintended, if welcome, consequence.

In addition to misinterpreting the meaning of our labels, participants may also be confused about what the labels imply about the recommended balance of diet and exercise. We do not want to imply that all calories consumed must be expended with physical activity to maintain a healthy energy balance. Our objective was to provide a better format than simple calorie counts for contextualizing energy content of different foods. Through this study, we hoped to gather qualitative data to guide effective application of our labels either as an addition to or replacement for numerical calorie information.

## Conclusions

Limitations of the current study include limited generalizability of our findings, and inability of the current study to predict how our labels might affect real world behavior. We used convenience sampling to recruit an ethnically and socioeconomically diverse study population but recruited in a limited geographic area and excluded participants under 25 years of age. The vast majority (90%) of our study population had adequate health literacy, which makes it difficult to predict how those with lower health literacy, almost 50% of the general population, [33] would use the labels.

In using focus groups, our study goal was not to provide a quantitative assessment of these labels. Focus groups are a valuable tool for gathering the breadth of opinions on a subject [34]. Through open-ended questioning, we were able to gather verbatim interpretation of various labels without prompting and report conveyed meaning. This helped us improve the label design and create labels that could be widely understood. However, we did not test label performance or compare our labels to other formats. We are interested in designing a physical activity equivalent label to compliment or replace calorie information on quick-service restaurant menus, but our study does not provide sufficient information on whether or how consumers might use physical activity equivalent labels. Moreover, it cannot predict whether these labels positively affect consumer choice behavior or the prevalence of overweight and obesity.

Our labels present a universally applicable physical activity equivalent for caloric information and do not account for several variables—age, gender, body weight—important to determining the actual metabolic results for an individual trying to use the label. Thus the labels may be overly simplistic and could cause confusion for consumers trying to offset caloric intake with physical activity.

Future studies should test these labels with a wider population that can provide both qualitative and quantitative data. A companion study is currently in press [35] in which we used a web-based survey to randomize participants to different label types and test performance with simulated food choices. Using this data, we anticipate finalizing adaptations to the label format and performing laboratory-based, randomized studies of consumer behavior using menus with a variety of label types, like those that have been used to test calorie menu labels [14,16]. Because laboratory-based studies are poorly generalizable to real world behavior, large-scale studies implementing different label types in quick-service restaurants will be necessary to fully measure their effects on the prevalence of overweight and obesity. Such studies would require governmental and industry participation.

Public health advocates and policy-makers need more and better information as they promote and implement various tools to combat overweight and obesity. Calorie menu labeling is an attractive option in that it provides information a majority of consumers want [25,36]. To have a positive effect on the prevalence of obesity, consumers would have to respond to new menu labels by purchasing and consuming fewer calories, eating more at home, patronizing restaurants that provide lower-calorie options, or exercising. The food service industry might also respond, as it did when the government expanded nutrition labeling laws on packaged products in the 1990s, by improving the nutritional profile of its offerings [17]. Early studies of calorie menu labels indicate they may not be having their intended effect [5,6].

Physical activity equivalent labels have the potential to be more persuasive than calorie information alone because they contextualize the information in familiar terms. Labels that make it easier to compare items on a menu facilitate better choices. Our label is designed to help people eat less and also encourage them to move more, a more complete approach to combat overweight and obesity than labels that only address nutritional content. More research is needed to establish whether consumers can and will apply such information in a way that leads to healthier living.

## Appendix 1: Focus group guide

Project: Design nutrition labels that use physical activity to contextualize energy information. For example, we will have a picture of a running person with a statement “You would have to run 50 minutes to burn the calories in this sandwich.” The focus group will be used to get consumer reactions to label design, refine the design, and get an idea of whether consumers understand the labels.

Questions:

1. Icebreaker: Where do you get your groceries?

2. How do you pick what food you eat?

3. How do you decide what foods are healthy or nutritious?

4. Think back to the last time you were at a fast food restaurant like (McDonalds, Burger King or Subway) ordering from the menu. How did you decide what to order? At this point, we will distribute the labels to the group. We may have a large poster of the labels so that we can talk about them. We also may put them on actual packaged food items. We will include several of our exercise label formats and one modeled after current calorie labels on menus.

5. a. Describe what symbol A means.

b. Describe what symbol B means.

c. Describe what symbol C means.

6. Would you change anything on this label?

-Which label do you like better? SHOW EXAMPLE  OF  LABEL  THAT  EXPRESSES ENERGY  INFORMATION  IN  DISTANCE (MILEAGE) RATHER THAN TIME

-Which label do you like better? SHOW EXAMPLE OF LABEL WITH DIFFERENT FONT

-Which label do you like better? SHOW EXAMPLE  OF  LABEL  WITH  DIFFERENT PICTURE

-Which label do you like better? SHOW EXAMPLE OF LABEL THAT HAS AVERAGE RECOMMENDED DAILY EXERCISE

7. Imagine yourself back at a fast food restaurant. If you had one of these labels in front of you, how would it affect what you ordered?

8 Imagine these labels on a cereal box at a grocery store. What label or information would help you choose between types of cereal?

9. We wanted you to help us evaluate and improve nutrition labels so that they are more useful for consumers. Do you have any ideas for nutrition labels that you would like to see? Is there anything about nutrition labels that you have not had a chance to say?

## Appendix 2: Sample calculations for menu labels

Example: Subway Ham Sandwich = 290 kcal

Energy expenditure for walking labels:

Walking at 30 min/mile pace = 3.2 kcal/min (for 160 lbs adult)

290 *kcal***/** 3.2 *kcal/min* = 91 *min* = 1.5 *hrs*

91 *min***/** 30 *min/mile* = 3 *miles*

Energy expenditure for running labels:

Running at 10 min/mile pace = 12.8 kcal/min (for 160 lbs adult)

290 *kcal***/** 12.8 *kcal/min* = 23 *min* = 0.4 *hrs*

23 *min***/** 10 *min/mile* = 2.3 *miles*

## Competing interests

The authors declare that they have no competing interests or financial disclosures.

## Authors’ contributions

JS designed the focus group format, facilitated the focus groups, took the lead on transcript analysis, and prepared the manuscript. SD helped facilitate focus groups, assisted in transcription, assisted in analysis, and edited the manuscript. DB helped facilitate focus groups, helped with label design and to identify key background literature, and edited the manuscript. PM helped with analysis and edited the manuscript. AV assisted in designing the focus group format, helped with label design, guided analysis and edited the manuscript. All authors read and approved the final manuscript.

## Authors’ information

Author Jonas J Swartz was a student at the University of North Carolina Gillings School of Public Health at during the time this research was conducted.
